# A simple technique for the excision of cutaneous carcinoma: the round block purse-string suture

**DOI:** 10.1186/1477-7819-12-263

**Published:** 2014-08-20

**Authors:** Edoardo Raposio, Michele Antonacci, Giorgia Caruana

**Affiliations:** Department of Surgical Sciences, Plastic Surgeon Division, University of Parma, Via Gramsci 14, 43126 Parma, Italy; Cutaneous, Mininvasive, Regenerative and Plastic Surgery Unit, Parma University Hospital, Via Gramsci, 43126 Parma, Italy

**Keywords:** Dermatologic surgery, Purse-string suture, Reconstructive surgery, Round block suture, Surgical oncology

## Abstract

**Background:**

Purse-string suture is a simple technique that can be used to reduce the surface area of circular wounds in an effort to obtain minimal scarring. In this report, we provide evidence of the effectiveness of the purse-string suture as a stand-alone procedure that allows a permanent primary complete closure of small to moderate skin defects. The procedure is used primarily for the repair of skin defects due to cutaneous tumor excision in older patients.

**Methods:**

The purse-string suture is executed by using a 1-0 absorbable suture, always by exiting and reentering intradermally and never penetrating the epidermis, in a circumferential fashion.

**Results:**

The immediate postoperative folds flatten in about a 4-week postoperative time span, and the resulting scar is the smallest obtainable.

**Conclusions:**

The round block purse-string suture is a simple technique which allows complete closure of skin defects without importing tissue from a distance, and it can be particularly suitable for older patients because of their skin laxity.

## Background

The purse-string suture was first described in dermatologic surgery by Peled *et al*. [1]. It is a simple technique that can be used to reduce the surface area of circular wounds in an effort to obtain minimal scarring. Its use over the years has been associated with other techniques. Brady *et al*. [2] performed the purse-string suture to reduce both the longitudinal and transverse dimensions of the wound so that they could place a small skin graft to complete the reconstruction. Ciatti and Greenbaum [3] described the use of the purse-string suture in combination with side-to-side bilateral adjacent tissue transfer. Using this procedure, they succeeded in closing or reducing large facial defects. Lin and Li [4] described use of a double-purse-string suture as an adjunct to conventional vertical sutures. Cohen *et al*. [5] and Zhu *et al*. [6] described using a similar suture to achieve partial closure of cutaneous defects as an adjunct to extensive undermining. In this report, we propose the use of the purse-string suture as a stand-alone procedure that allows permanent primary complete closure of small to moderate skin defects, especially when side-to-side closure is not recommended due to a poor tissue laxity or epidermal thinning.

## Methods

**Figure 1:**
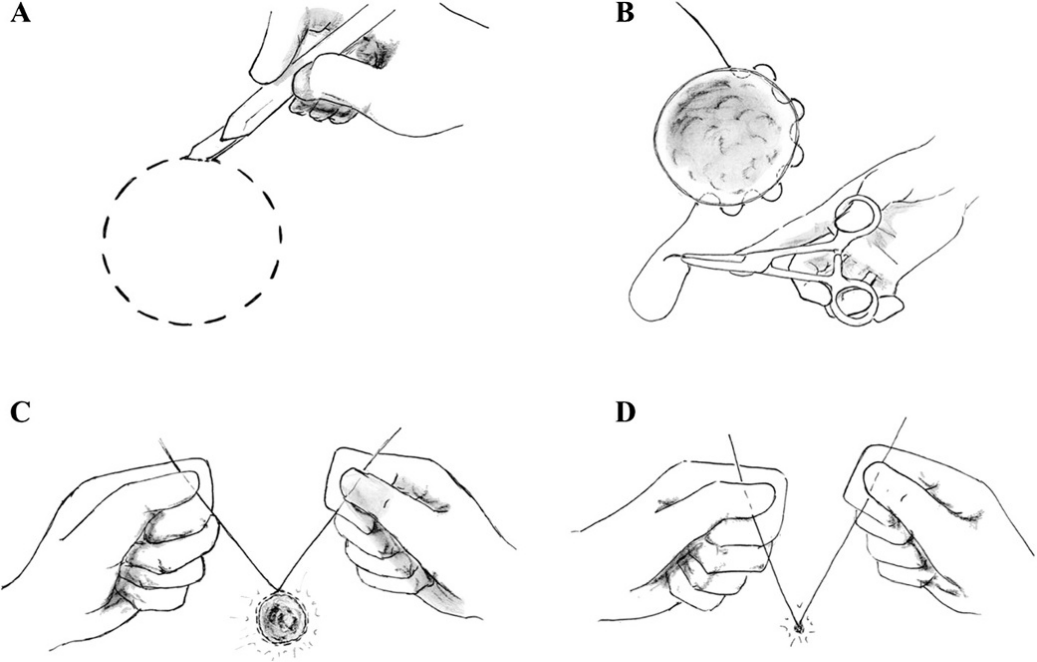
**Illustration depicting steps in the purse-string procedure. (A)** Round skin markings for surgical excision of the lesion. **(B)** Intradermal positioning of the purse-string suture in a circumferential fashion. **(C)** and **(D)** The suture was pulled and gently tied to obtain complete closure of the skin defect.

**Figure 2:**
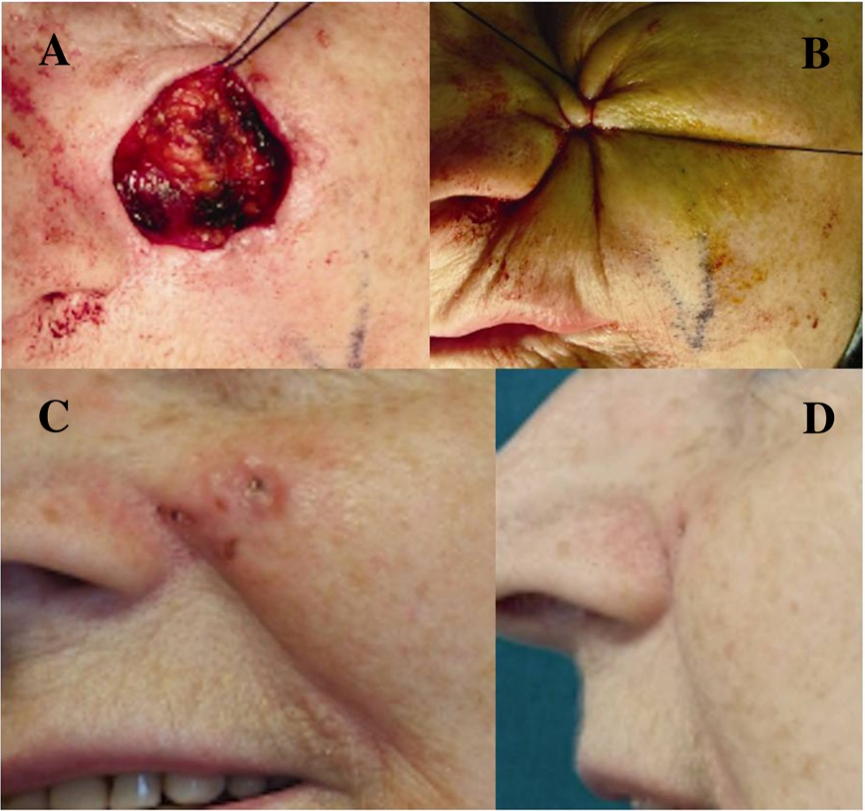
**Operative photographs of our patient, a 65-year-old woman with a basal cell carcinoma of the left paranasal cheek (1.6 cm diameter). (A)** Round surgical excision of the lesion. **(B)** Previously positioned suture is tied until complete closure is reached. **(C)** and **(D)** Photographs showing postoperative results 3 months after surgery.

After circular skin marking (Figure 1A), we performed, while the patient was under local anesthesia, a round surgical excision of the lesion (Figure 2A) with minimum (5 mm) subcutaneous undermining of the wound margins, thus decreasing the chances of bleeding complications and allowing maximum vascularity at the edges to be maintained [7, 8]. The purse-string suture was executed by using a 1-0 absorbable suture. The suture was exited and reentered, always intradermally and never penetrating the epidermis, in a circumferential fashion, with the needle always inserted about 2 mm from the dermal exit site (Figure 1B). This sequence was continued until the suture was passed all the way around the entire perimeter of the wound. When the initial entry and final exit points were met, the suture was pulled with increasing strength and then gently tied to obtain complete closure of the skin defect (Figures 2C, 1D and B). Once the closure was completed, the suture knot was tied within the wound. No external stiches were necessary.

## Results and discussion

The immediate postoperative period was characterized by surrounding skin distortion with the development of some concentric radial skin folds. Despite that, the folds flattened in about a 4-week postoperative time span. As such, the resulting scar was the smallest obtainable (Figure 2C and D).

Surgical excision of facial skin defects often produces deformities that are round in shape. Sometimes the closure can turn into a real challenge for surgeons, especially when attempting to minimize the scar. In selected cases, the purse-string suture provides complete wound closure, minimizing the dimensions of the resulting scar, which has been confirmed by data reported in the literature [7, 8]. Differently from the previously described techniques, there was no need to remove any suture after the surgery as we did not use external stiches or nonabsorbable sutures; thus the patient was spared this inconvenience. Using circumferential tissue advancement, this procedure is primarily suited for the repair of skin defects due to cutaneous tumor excision in older patients. The extensibility and laxity of the skin in these patients adapt nicely, allowing complete closure of defects without the need to import tissue from a distance. The minimal undermining required allows the scar to mature along the physiological Langer’s lines of the skin, preserving the local skin perfusion.

## Conclusions

In our patient, the round block purse-string suture proved to be a simple and rapid technique for the excision of cutaneous carcinoma. It allowed a definitive round closure after tumor removal, leaving the smallest possible scar. This closure is best suited to the repair of cutaneous defects in the cervicofacial area; however, it should not be performed in close proximity to free margins (that is, the ocular and buccal regions) to avoid permanent distortion of these structures.

### Consent

Written informed consent was obtained from the patient for the publication of this report and any accompanying images.
